# Geospatial analysis of determinants of neonatal mortality in Ghana

**DOI:** 10.1186/s12889-021-10473-w

**Published:** 2021-03-12

**Authors:** Duah Dwomoh

**Affiliations:** Department of Biostatistics, School of Public Health, College of Health Sciences, University of Ghana, Legon, Accra, Greater Accra Ghana

**Keywords:** Neonatal mortality, Child health, Geospatial modeling, Ghana

## Abstract

**Background:**

Ghana did not meet the Millennium Development Goal 4 of reducing child mortality by two-thirds and may not meet SDG (2030). There is a need to direct scarce resources to mitigate the impact of the most important risk factors influencing high neonatal deaths. This study applied both spatial and non-spatial regression models to explore the differential impact of environmental, maternal, and child associated risk factors on neonatal deaths in Ghana.

**Methods:**

The study relied on data from the Ghana Demographic and Health Surveys (GDHS) and the Ghana Maternal Health Survey (GMHS) conducted between 1998 and 2017 among 49,908 women of reproductive age and 31,367 children under five (GDHS-1998 = 3298, GDHS-2003 = 3844, GDHS-2008 = 2992, GDHS-2014 = 5884, GMHS-2017 = 15,349). Spatial Autoregressive Models that account for spatial autocorrelation in the data at the cluster-level and non-spatial statistical models with appropriate sampling weight adjustment were used to study factors associated with neonatal deaths, and a *p*-value less than 0.05 was considered statistically significant.

**Results:**

Population density, multiple births, smaller household sizes, high parity, and low birth weight significantly increased the risk of neonatal deaths over the years. Among mothers who had multiple births, the risk of having neonatal deaths was approximately four times as high as the risk of neonatal deaths among mothers who had only single birth [aRR = 3.42, 95% CI: 1.63–7.17, *p* < 0.05]. Neonates who were perceived by their mothers to be small were at a higher risk of neonatal death compared to very large neonates [aRR = 2.08, 95% CI: 1.19–3.63, *p* < 0.05]. A unit increase in the number of children born to a woman of reproductive age was associated with a 49% increased risk in neonatal deaths [aRR = 1.49, 95% CI: 1.30–1.69, *p* < 0.05].

**Conclusion:**

Neonatal mortality in Ghana remains relatively high, and the factors that predisposed children to neonatal death were birth size that were perceived to be small, low birth weight, higher parity, and multiple births. Improving pregnant women’s nutritional patterns and providing special support to women who have multiple deliveries will reduce neonatal mortality in Ghana.

**Supplementary Information:**

The online version contains supplementary material available at 10.1186/s12889-021-10473-w.

## Background

Globally, neonatal deaths remains a major public health concern as 5.3 million children under age five died in 2018 [1]. The risk of a family having or experiencing neonatal deaths within the first 30 days of the child life is still highest in the World Health Organization (WHO) African Region (76 per 1000 live births), which is approximately eight times as high as the WHO European Region (9 per 1000 live births) [1]. For instance, in 2018 alone, the under-five mortality rate in low-income countries was 68 deaths per 1000 live births, which is almost 14 times the average rate in high-income countries (5 deaths per 1000 live births) [1]. Neonatal mortality remains a major contributor to under-five mortality [2].

Despite the huge investment made by the governments in sub-Saharan Africa and foreign partners towards reducing neonatal deaths in Sub-Saharan Africa, only 12 countries in the World Health Organization’s African Region met the Millennium Development Goal 4 (MDG4) to reduce U5MR by two-thirds by 2015. These countries are Eritrea, Ethiopia, Liberia, Madagascar, Malawi, Mozambique, Niger, Rwanda, Senegal, Tanzania, Uganda, and Zambia [3].

One of the Sustainable Development Goals 3 (SDG 3) primary objectives is to reduce neonatal mortality to at least as low as 12 per 1000 live births [4]. However, the rate of progress toward these goals significantly varied at the national level, demonstrating an essential need for tracking even more local trends in child mortality [5].

Global estimates from 2010 show that approximately 40% of all deaths to children under age 5 occur during the neonatal period [6]. Many countries have made progress in reducing deaths among children under age 5, but these gains have been predominantly among children age 1–4 [7]. Little progress has been made in reducing the mortality risk for children under age 12 months, especially for neonates, in the first month of life [7].

Neonatal mortality rates in Ghana remain unacceptably high despite the substantial financial investment in the health sector over the last decade. Ghana’s neonatal death rate fell gradually from 202.8 deaths per 1000 live births in 1968 to 49.3 deaths per 1000 live births in 2017. To meet the set target of sustainable development goal 3 (SDG 3), there is the need to understand complex causes of neonatal deaths [8]. To improve neonates’ health, it is necessary for decision-makers to effectively comprehend the correlation between maternal, child, and household characteristics and how they co-integrate with geospatial factors to influence the survival probability of neonates in Ghana. Policymakers’ ability to understand the variation in neonatal mortality and the underlying causes is important for targeted intervention [9]. Recently, Grady et al. [2] have used geographically weighted Poisson regression models to capture the spatially varying relationships between neonatal mortality and the following indicators: maternal education, women’s empowerment, home births, mothers without an education, and mothers whose husbands decided on contraceptive practices, rural residency.

Full-scale interventions are costly, especially in poverty-driven economies in Sub-Saharan Africa, and hence interventions must be proven to work, selective, and targeted at only essential risk factors associated with neonatal deaths [10]. With the adoption of the Sustainable Development Goals (SDGs) in 2015, which established ambitious targets for improving child survival by 2030, optimal intervention planning and targeting will require an understanding of factors influencing the high incidence of neonatal mortality [5]. This study aimed to use a more rigorous geospatial and traditional statistical modeling approach to identify key risk factors associated with neonatal deaths in Ghana to target interventions.

## Methods

### Data source

This study pooled data from the Ghana Demographic Health Surveys (GDHS) conducted in 1998/1999, 2003, 2008, and 2014, and Ghana Maternal Health Survey (GMHS) conducted in 2017. The GDHS is a nationally representative household survey of 24,846 women age 15–49 and an average response rate of 96.7%. The total number of children from the GDHS was 16,018. The GMHS interviewed 25,062 eligible women aged 15–49 in the selected households with an average response rate of 99.0%. With the GMHS, the study restricted the analysis to only children who were born alive in the 5 y preceding the survey. The two distinct surveys have variables on neonatal mortality and other relevant covariates of interest. The study relied on the 1998–2014 GDHS and 2017 GMHS, the latest survey conducted in Ghana that has data on neonatal mortality and relevant predictors. The 2017 GMHS was analyzed independently from the GDHS datasets. The analysis was restricted to only women who had children in the last 5 y preceding the survey. The sample contains the total number of children under-five born in the 5 y preceding the survey, and that data contains the information on their respective mothers and households in terms of education, place of residence, household wealth, parity, household size, etc.

A detailed description of the data sets used for the study can be found in Table 1.

**Table 1:** Description of the data source used for the study

Year of survey	Source of data	Number of women aged 15–49 years interviewed	Number of children born in the five years preceding each survey	The average response rate of eligible women (%)
1998	GDHS	4843	3298	97.4
2003	GDHS	5691	3844	95.7
2008	GDHS	4916	2992	96.5
2014	GDHS	9396	5884	97.3
2017	GMHS	25,062	15,349	99.0
Total		49,908	31,367	Average response rate = 97.2%

*Abbreviations*: *GDHS* Ghana Demographic and Health Survey, *GMHS* Ghana Maternal Health Survey

### Sampling design

The 1998–2017 GDHS and GMHS were based on a multi-stage stratified cluster sampling. First, the country was stratified by the 10 regions and urban and rural areas, generating 20 sampling strata. Samples of enumeration areas (EAs) were selected independently in each stratum in two stages. In the first stage of obtaining the samples, EAs (residential households) were selected with probability proportional to the EA size (number of residential households) and with independent selection in each of the 20-sampling stratum. The EA size was obtained from the 2010 Population and Housing Census. The sampling frame for the survey was obtained by conducting a household listing operation in all the selected EAs, and the resulting lists of households served as a sampling frame for the selection of households in the second stage. In the second stage of selection, a fixed number of households per cluster (EA) was selected with an equal probability systematic selection from the newly created household listing. The trained interviewers visited and interviewed only the selected households. The detailed sampling design and sample selection for the 2014 GDHS can be found in [11].

### Outcome measure

This study investigated neonatal deaths in Ghana. All the children who died within the 5 y but the death that occurred within the first 28 days after delivery were classified as neonatal deaths. The neonatal mortality rate was estimated within 5 y preceding the survey and estimated using the synthetic cohort probability method.

### Predictors of neonatal deaths

This study relied on the Mosley and Chen conceptual framework for neonatal deaths in developing countries [12].

### Characteristics of the household

The household characteristics that were studied include place of residence, region, zone (coastal, middle, northern), age of the household head, sex of the household head, type of oil the household mainly use for cooking, whether the household has access to the internet, number of household members covered with health insurance, number of sleeping rooms, whether the household has electricity, main floor material of the household, main roofing material, main wall material, type of cooking fuel, the frequency at which household members smoke inside the house, place where household members wash their hands, place of cooking, access to an improved water source and household access to an improved sanitation facility, number of women aged 15–49 years in the household, whether the household has iodized salt, whether any member of the household own any agricultural land, whether the household has any livestock, herds, other farm, animals, or poultry, and household wealth**.** The wealth index was used as a proxy to measure the socioeconomic status of the household. The composite wealth index constructed by the DHS is based on household-level data on assets, services, and amenities and ranks households according to their wealth level. A household is defined as having access to an improved water source if it has any of the following: piped water into the dwelling, yard, or plot; public tap or standpipe, tubewell, or borehole; a protected dug well or protected spring; rainwater; or bottled water. Studies have found access to an improved water source to be associated with infant survival [13]. A household is defined as having improved sanitation if it has any of the following types of facilities: a flush or pour-flush to a piped sewer system, septic tank, or pit latrine; a ventilated improved pit latrine; a pit latrine with a slab; or composting sanitation. Household access to improved sanitation is associated with lower levels of infant mortality [13]. Finally, this study investigated the impact of household size on neonatal deaths.

### Lifestyle factors

In the last seven days, the number of fruits and vegetable consumption was used as a proxy to assess fruits and vegetable consumption during the antenatal period.

### Characteristics of the child

The characteristics of the child that were studied include sex of the child (male or female), the weight of the child at birth, multiple births (single or multiple birth), birth order, birth spacing. The birth order was grouped into four categories: first births, second births, third births, and fourth or higher-order births. The birth spacing was grouped into three categories: intervals of less than 24 months, 24–35 months, and 36 or more months, similar to what has been reported by Pezzullo et al.,2016 [10].

### Maternal factors

The following maternal related factors were studied: the current age, age at first birth, parity, the current marital status, number of unions, religion, ethnicity, highest educational level of the mother, mothers body mass index, whether the mother has ever terminated pregnancy, currently using a contraceptive, heard about family planning method, ever been diagnosed with hypertension, anemia, the ideal number of children, owns of a house or land, level of autonomy, level of violence against women and mothers stature. Short stature is an indication of the mother’s nutrition status from the fetal period through adolescence and reflects the magnitude of environmental exposures such as infection, illness, and economic hardship during this period. Some studies have found a positive correlation between neonatal mortality and a mother’s stature [14]. We also studied the partner/husband’s characteristics in the context of their educational level and occupation.

### Other factors

The following represent the characteristics of maternal and delivery care and coverage of other interventions that may have influenced neonatal deaths over the study period: the place of delivery (home or hospital), type of delivery (normal or cesarian section), whether the child or the mother was covered by health insurance, early initiation of breastfeeding, currently breastfeeding, health-seeking behavior (visited health facility in the last six months), the presence of a program helping women to access health services, dwelling sprayed in the last 12 month, household ownership of bednet, number of malaria messages heard, heard about TB, heard messages about antenatal care, mothers slept under-bednet the previous night, children under-five that slept under a bednet, number of mosquito bednet in the household, whether mother received tetanus injection, iron and Fansidar (sulfadoxine and pyrimethamine) for malaria prevention during pregnancy, antenatal care attendance. Admittedly, household ownership of bednet was measured at the time of the survey; however, we use this information as a proxy for mosquito net ownership during the pregnancy. During pregnancy, malaria can cause intrauterine growth retardation and preterm delivery, leading to low birth weight and neonatal death [15]. Besides, mothers who slept under-bednet the previous night were used as a proxy for the mother’s net use during her pregnancy and during the neonatal period. Early initiation of breastfeeding could be associated with neonatal survival [16]. In summary, most of the predictors in this study were used as a proxy to understand the characteristics of the respondents before pregnancy, during pregnancy, and after delivery.

### Geospatial covariates

Studies have linked climatic and environmental factors such as precipitation, vegetation density, and length of growing season with the prevalence of vector-borne diseases such as malaria because these factors are related to favorable habitats for disease vectors [17]. The following geospatial covariates: aridity, built population, drought episodes, enhanced vegetation index, global human footprint, night light composite, malaria, insecticide-treated net (ITN) coverage, proximity to national borders, proximity to a protected area, proximity to water, rainfall, growing season length, environmental temperature, travel time, slope and livestock density were obtained from the Spatial Data Repository under the DHS program (https://spatialdata.dhsprogram.com/covariates/). The definition of the geospatial covariate can be found in Appendix 4 of supplementary material.

### Statistical methods

The study employed two main statistical techniques (spatial and non-spatial analyses) to explore the risk and protective factors of neonatal deaths in Ghana.

### The non-spatial statistical methods-traditional regression models

In this study, the unit of analysis is all children born in the five years preceding each survey (1998, 2003, 2008, 2014, and 2017).

The traditional regression models assume that both geospatial and non-geospatial covariates are identically and independently distributed over the geographical space, although all statistical analyses adjusted for weighting, clustering, and stratification at the individual-level analysis. The study employed a three-stage statistical analytic technique. First, since the study pooled data from different DHS at different survey periods in Ghana, the women’s standard weights were de-normalized to obtained one pooled data set for all the GDHS (1998–2014). Second, bivariate analysis of the selected predictors (household, maternal, child and interventions) of neonatal deaths was conducted using the Rao-Scott designed based adjusted Chi-square test of independence due to the correlation among units within the same cluster{Rao, 1981 #56}. Third, the study assessed each covariate’s effect by fitting a Poisson model that includes the covariate of interest and the year of the survey fixed effect. Finally, nine different Poisson regression models were fitted to assess the multivariable effect of the predictors on neonatal deaths as follows. Model 1 assessed the joint effect of all household factors on neonatal deaths. Model 2 and 3 assessed the effect of only maternal and child-related factors respectively. Model 4 evaluated the effect of household and child factors. Model 5 assessed the combined effect of household and maternal factors. Model 6 has a combination of maternal and child factors. Model 7 assessed the joint effect of household, maternal, and child-related characteristics.

Model 8 has only environmental factors (geospatial covariates), and this includes vegetation index, population density, malaria prevalence per cluster, environmental temperature, rainfall pattern, the length of the growing season, goat livestock, and proximity to a protected area. The 9th model integrates household, maternal, child, and environmental factors.

The study assessed the performance of these nine models for predicting neonatal deaths by estimating the Schwarz’s Bayesian Information Criteria (BIC), Akaike Information Criterion (AIC), and a 10-fold cross-validated area under the receiver operating characteristic curve (cvAUROC). The best model was selected based on the subject matter knowledge and the estimates from three model performance metrics (AIC, BIC, cvAUROC). The study excluded the 1998 GDHS data set from all the multivariable models since the survey did not measure most of the important predictors of interest. For instance, household wealth, which was used as a proxy to measure socioeconomic status was not measured in the 1998 GDHS, and including the data would have reduced the effective sample size substantially.

### The spatial statistical methods: assessing the effect of geospatial covariates on neonatal deaths

This study performed a descriptive cluster-level analysis and spatial distribution of neonatal deaths by aggregating the variables from household survey data and geospatial covariates collected by the DHS program to the cluster level for 2003–2014. Sampling weights were applied to calculate appropriate estimates for each indicator at the cluster level. The units of analysis were clusters of households between 2003 and 2014. In all, the study analyzed 1245 unique clusters between 2003 and 2014. To determine whether there was a need for applying Autoregressive Spatial Models that account for spatial autocorrelation in the data, we first tested the assumption of spatial autocorrelation using the Moran I index. None of the geospatial covariates showed any form of spatial autocorrelation (Additional file 1: Appendix B1). Each geospatial covariate was independently evaluated to assess how they contributed to the final baseline model’s overall discriminating ability using cvAUROC. This was achieved by estimating the traditional modified negative binomial regression model that adjusts for weighting, clustering, and stratification. All continuous geospatial covariates were fitted via a four-knot restricted cubic spline functions. The study assessed the covariates’ multicollinearity using the variance inflation factor (VIF) (Additional file 1: Appendix B2). All statistical analyses were conducted using Stata 15 (StataCorp, College Station, Texas, USA), and *p*-values less than 0.05 were considered statistically significant.

## Results

### Descriptive statistics

The average age of the head of the household was approximately 40 ***±***11.02 years (range = 16–99 years), and females headed 28% of the households with an average household size of 5.7. The percentage of households in rural areas was approximately 64%. The percentage of male and female children born in the 7 y preceding each survey was almost equal (males = 51.1%, females = 49%). The prevalence of multiple births was 4.5, and 3.7% of the children had a low birth weight. The percentage of 4 or more antenatal visits was 76.1, and 41% of the mothers did not deliver in a recommended health facility (home delivery). Seven percent of all the deliveries were conducted through a cesarian section, and 48.2% of the women breastfed after 1 h of delivery. Children born within the optimal 24–35-month birth spacing was 26%.

### The trend of neonatal mortality in Ghana: 1998–2017

**Fig. 1:**
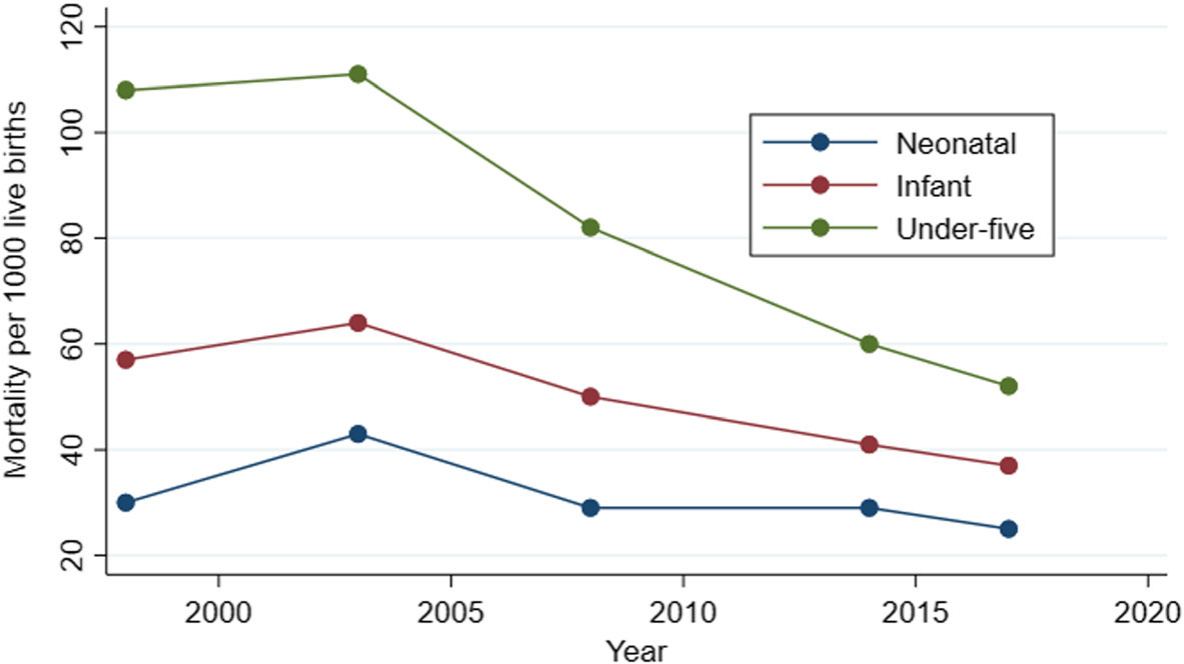
Mortality trends in Ghana between 2003 and 2014

There has been a steady decline in neonatal deaths between 1998 and 2017 over the past 15 years. Specifically, neonatal mortality declined from 30 per 1000 live birth in 1998 to 25 per 1000 live births in 2017 for all births in the 5 y preceding the survey, although there were some fluctuations between 2003 and 2014. Figure 1 provides a brief summary of mortality trends in Ghana between 2003 and 2014.

### Factors that independently influenced neonatal mortality

The only household factor that influences the risk of neonatal deaths was the household size. Neonatal mortality was higher among households with only a few members (4 or less). The risk of neonatal mortality reduced by 29 percentage points among households with eight or more members compared to a household with four or less number of inhabitants [adjusted relative risk; aRR = 0.7, 95% CI:0.5–0.9; *p* < 0.05, Additional file 1: Appendix 1A]. The risk of having neonatal deaths among mothers who had multiple births was approximately five times as high as the risk of neonatal deaths among mothers who had single births [aRR = 4.5, 95% CI: 3.25–6.49, *p* < 0.01, Additional file 1: Appendix 1A]. The risk of neonatal mortality among mothers who gave birth to children in less than 24 months after the previous childbirth was approximately two times the risk of neonatal deaths if the index baby is the first child of the mothers [aRR = 1.7, 95% CI: 3.3–6.5, *p* < 0.001, Additional file 1: Appendix 1A]. Neonates who were perceived to be small were at a higher risk of neonatal mortality [aRR = 1.7, 95% CI: 1.2–2.5, *p* < 0.01, Additional file 1: Appendix 1A]. The risk was reduced by 32% among mothers who received two or more tetanus injections than mothers who received no injection [aRR = 0.7, 95% CI: 0.5–0.9, *p* < 0.05, Additional file 1: Appendix 1A]. Women who were delivered using cesarean section were at a higher risk of having neonatal mortality compared to women who had vaginal delivery [aRR = 1.7, 95% CI: 1.2–2.5, *p* < 0.01, Additional file 1: Appendix 1A]. Mothers who gave birth after age 30 had a higher risk of experiencing neonatal mortality compared to those who are less than 18 years [aRR = 1.9, 95% CI: 1.1–3.2, *p* < 0.01, Additional file 1: Appendix A1]. Mothers who had five or more children were at a higher risk of neonatal mortality compared to mothers with only one child [aRR = 1.6, 95% CI: 1.1–2.2, *p* < 0.01, Additional file 1: Appendix A1]. Children born into families where the biological father has more than one wife were also at a greater risk of neonatal deaths [aRR = 1.4, 95% CI: 1.1–1.8, *p* < 0.01, Additional file 1: Appendix A1]. The use of contraceptives among women reduced the risk of neonatal mortality by 25 percentage points compared to women who do not use any form of contraceptive [aRR = 0.8, 95% CI: 0.6–1.0, p < 0.01, Additional file 1: Appendix A1]. The stature of a woman could either be genetic or reflective of long term exposure to poor diet. Tall women (within the 75th percentile of height) were at a lower risk of having neonatal deaths compared to short women (within the 25th percentile of height distribution) [aRR = 0.7, 95% CI: 0.5–0.9, p < 0.01, Additional file 1: Appendix A1].

### Comparative analysis of nine models for neonatal mortality 2003–2014

Nine different models were built and evaluated to assess how they explain neonatal mortality dynamics in Ghana. The nine models’ construction was to enable us to evaluate the statistical significance of one set of independent variables whiles simultaneously controlling for another set of independent variables.

Table 2 provides a summary of the nine models for neonatal deaths. Although these models’ discrimination ability was not substantial, maternal and child factors virtually explain the bulk of the variations in neonatal deaths (neonatal; cvAUROC = 73%; Table 2). Clearly, the household factors and the geospatial covariates (environmental factors) alone did not explain much of child mortality variations. It presupposes that more emphasis should be placed on addressing maternal and child factors. However, models that integrate household, maternal, child, and environmental factors had a minimum bias in all cases (smallest BIC and AIC). We applied subject matter knowledge in choosing the final model, coupled with the AIC, BIC, and cvAUROC estimates. Model 9 was selected as the best model to predict the risk of neonatal mortality in Ghana.

**Table 2:** Evaluating the predictive performance of the nine models for neonatal mortality: 2003–2014 Ghana Demographic and Health Surveys

Models	variables	Neonatal Mortality		
Adjusted for all models	Persons year at risk, and year of survey	AIC	BIC	cvAUROC
Model 1 = Household factors	Age of the household head, sex of the household head, place of residence, household size, zone location, access to an improved water source, access to improved water facility, household ownership of bed-net	8,390,012	8,390,131	58.0%
Model 2 = Child characteristics	Sex of the child, multiple births, birth order, birth spacing, size of the baby at birth	7,709,512	7,709,616	66.0%
Model 3 = Maternal factors	Mothers age at birth, marital status, literacy, parity, type of delivery, place of delivery, early breastfeeding, contraceptive use, history of terminating a pregnancy	5,761,778	5,761,889	61.0%
Model 4 = Model 1 + Model 3	Household factors + Maternal factors	5,586,465	5,586,672	63.0%
Model 5== Model 1 + Model 2	Household factors + Child characteristics	7,528,049	7,528,250	67.0%
Model 6 = Model 2 + Model 3	Child +Maternal factors	5,335,693	5,335,886	73.0%
Model 7 = Model 1 + Model 2+ Model 3	Household factors + Maternal factors+ Child factors	5,156,220	5,156,509	73.0%
Model 8 = Geospatial covariates	The length of the growing season in months, environmental temperature, population density, the prevalence of malaria, proximity to a protected area, net vegetation index, livestock goat.	7,663,200	7,663,348	54%
Model 9 = Model 7+ Model 8	Household factors + Maternal factors+ Child factors+ Geospatial covariates	4,589,203	4,589,614	74%

*Abbreviation*: *AIC* Akaike Information Criterion, *BIC* Bayes Information Criterion, *cvAUROC* Cross Validated Area Under the Receiver Operating Characteristics Curve

### Multivariable regression analysis of factors associated with neonatal mortality: evidence from the pooled Ghana Demographic and health surveys (2003–2014)

The findings from the pooled data set from 2003 to 2014 GDHS, including the environmental factors, revealed that household size, multiple births, perceived birth weight, parity, and use of contraceptives influenced neonatal deaths. An increase in the household size reduced neonatal deaths by 18% [aRR = 0.82, 95% CI: 0.73–0.92, *p* < 0.05, Table 3]. Among mothers who had multiple births, the risk of having neonatal deaths is approximately four times as high as the risk of neonatal deaths among mothers who had only single birth [aRR = 3.42, 95% CI: 1.63–7.17, *p* < 0.05, Table 3]. Neonates who were perceived by their mothers to be small were at a higher risk of neonatal death compared to very large [aRR = 2.08, 95% CI: 1.19–3.63, *p* < 0.05, Table 3]. A unit increase in the number of children born to a woman of reproductive age is associated with a 49% increased risk in neonatal deaths [aRR = 1.49, 95% CI: 1.30–1.69, *p* < 0.05, Table 3]. The use of contraceptive reduced the risk of neonatal deaths by 46% [aRR = 0.56, 95% CI: 0.35–0.90, *p* < 0.05, Table 3]. On average, a one-unit increase in the log-population density is equivalent to a 24% reduction in neonatal deaths [aRR = 0.76, 95%CI: 0.60–0.96; *p* < 0.05, Table 3].

**Table 3:** Multivariable regression analysis of factors associated with neonatal mortality: Evidence from the pooled Ghana Demographic and Health Surveys (2003–2014)

Variables	MODEL 1	MODEL 2	MODEL 3	MODEL 4	MODEL 5	MODEL 6	MODEL 7	MODEL 8	MODEL 9
	aRR [95% CI]	aRR [95% CI]	aRR [95% CI]	aRR [95% CI]	aRR [95% CI]	aRR [95% CI]	aRR [95% CI]	aRR [95% CI]	aRR [95% CI]
**HOUSEHOLD FACTORS**									
**Age of the household head**	1.01 [1.01–1.02]**			**0.99 [0.97–1.00]**	1.02 [1.01–1.02]**		**1.00 [0.99–1.02]**		**1.00 [0.99–1.02]**
**Sex of household head**									
Male	**1**			**1**	**1**		**1**		**1**
Female	**0.98 [0.74–1.29]**			0.62 [0.40–0.97]*	**0.90 [0.67–1.22]**		**0.64 [0.40–1.03]**		**0.76 [0.49–1.18]**
**Place of residence**									
Urban	**1**			**1**	**1**		**1**		**1**
Rural	**1.44 [1.00–2.08]**			**1.24 [0.77–2.00]**	**1.35 [0.92–1.99]**		**1.25 [0.74–2.09]**		**1.39 [0.81–2.39]**
**Zone**									
Coastal	**1**			**1**	**1**		**1**		**1**
Southern	**1.19 [0.89–1.6]**			**0.93 [0.67–1.29]**	**1.09 [0.81–1.48]**		**0.87 [0.61–1.23]**		**0.76 [0.44–1.32]**
Northern	**1.25 [0.85–1.84]**			**1.15 [0.73–1.83]**	**1.28 [0.85–1.94]**		**1.20 [0.71–2.02]**		**1.35 [0.49–3.72]**
**Household size**	0.90[0.85–0.97]**			0.85 [0.76–0.94]**	0.83 [0.76–0.92]***		0.80 [0.71–0.9]***		0.82 [0.73–0.92]**
**Wealth index**									
Poorest	**1**			**1**	**1**		**1**		**1**
Poorer	**0.83 [0.57–1.21]**			**0.77 [0.51–1.15]**	**0.86 [0.59–1.27]**		**0.80 [0.52–1.22]**		**0.92 [0.60–1.40]**
Middle	**1.17 [0.74–1.84]**			**0.97 [0.56–1.67]**	**1.09 [0.67–1.78]**		**0.97 [0.54–1.75]**		**1.1 [0.60–2.01]**
Richer	**0.99 [0.56–1.75]**			**0.71 [0.36–1.42]**	**0.93 [0.51–1.71]**		**0.72 [0.34–1.51]**		**0.81 [0.37–1.80]**
Richest	**1.15 [0.63–2.12]**			**0.96 [0.43–2.13]**	**0.84 [0.43–1.65]**		**0.77 [0.33–1.82]**		**0.98 [0.38–2.54]**
**Household access to an improved water source**									
Improved	**1**			**1**	**1**		**1**		**1**
Unimproved	**1.07 [0.77–1.47]**			**0.98 [0.67–1.44]**	**0.95 [0.67–1.36]**		**0.87 [0.57–1.34]**		**0.88 [0.57–1.34]**
**Household access to an improved sanitation facility**									
Improved	**1**			**1**	**1**		**1**		**1**
Unimproved	**0.78 [0.56–1.08]**			**0.85 [0.58–1.24]**	**0.73 [0.52–1.05]**		**0.73 [0.47–1.13]**		**0.65 [0.41–1.02]**
**Have bed net**									
No net	**1**			**1**	**1**		**1**		**1**
Have net	**0.98 [0.72–1.33]**			**0.89 [0.62–1.27]**	**1.05 [0.75–1.47]**		**0.88 [0.60–1.29]**		**0.95 [0.63–1.43]**
**CHILD FACTORS**									
**The sex of the child**									
Male		**1**			**1**	**1**	**1**		**1**
Female		**0.82 [0.64–1.04]**			**0.83 [0.65–1.06]**	0.75 [0.56–1]*	0.74 [0.55–1]*		**0.72 [0.53–1.00]**
**Multiple births**									
Single birth		**1**			**1**	**1**	**1**		**1**
Multiple births		4.10 [2.59–6.49]***			4.99 [3.01–8.28]***	3.29 [1.73–6.26]***	3.88 [1.96–7.68]***		3.42 [1.63–7.17]**
**Birth order**									
1st child		**1**			**1**	**1**	**1**		**1**
2nd child		**0.39 [0.08–2.00]**			**0.36 [0.07–1.88]**	**0.43 [0.08–2.38]**	**0.4 [0.07–2.31]**		**0.5 [0.09–2.91]**
3rd child		**0.47 [0.09–2.48]**			**0.46 [0.08–2.54]**	**0.39 [0.07–2.26]**	**0.38 [0.06–2.38]**		**0.46 [0.07–2.94]**
4th + child		**0.55 [0.1–2.94]**			**0.62 [0.11–3.41]**	0.14 [0.02–0.84]*	0.14 [0.02–0.91]*		**0.17 [0.03–1.09]**
**Birth spacing**									
First child		**1**			**1**	**1**	**1**		**1**
< 24 months		**3.25 [0.62–16.97]**			**3.95 [0.74–21.15]**	**2.16 [0.35–13.25]**	**2.3 [0.36–14.59]**		**2.02 [0.32–12.75]**
24–35 months		**1.66 [0.32–8.70]**			**2.00 [0.37–10.85]**	**1.14 [0.19–6.89]**	**1.23 [0.19–7.75]**		**0.84 [0.14–5.02]**
36+ months		**1.28 [0.25–6.49]**			**1.37 [0.26–7.21]**	**0.66 [0.12–3.74]**	**0.62 [0.10–3.68]**		**0.48 [0.08–2.87]**
**Perceived birth weight**									
Very large		**1**			**1**	**1**	**1**		**1**
Large		**0.75 [0.51–1.11]**			**0.78 [0.53–1.16]**	**0.8 [0.48–1.34]**	**0.8 [0.47–1.34]**		**0.88 [0.5–1.53]**
Average		**0.79 [0.53–1.18]**			**0.77 [0.51–1.16]**	**1.02 [0.6–1.73]**	**0.94 [0.54–1.63]**		**1.05 [0.58–1.90]**
Small		1.53 [1.03–2.26]*			**1.48 [0.99–2.2]**	1.85 [1.09–3.16]*	1.74 [1.02–3.00]*		2.08 [1.19–3.63]*
**MATERNAL FACTORS**									
**Mothers age at first-child**									
< 18			**1**	**1**		**1**	**1**		**1**
18–29			**1.03 [0.74–1.43]**	**1.11 [0.79–1.57]**		**0.93 [0.65–1.34]**	**0.98 [0.67–1.44]**		**1.15 [0.75–1.76]**
30+			**1.31 [0.55–3.11]**	**1.57 [0.65–3.82]**		**1.05 [0.42–2.62]**	**1.14 [0.44–2.92]**		**1.03 [0.36–2.91]**
**The current marital status of the mother**									
Never married			**1**	**1**		**1**	**1**		**1**
Married			**0.95 [0.59–1.54]**	**0.72 [0.42–1.26]**		**1.06 [0.64–1.77]**	**0.87 [0.48–1.55]**		**1.23 [0.67–2.25]**
Not married but living together			**0.87 [0.50–1.52]**	**0.68 [0.36–1.28]**		**0.89 [0.50–1.61]**	**0.71 [0.36–1.40]**		**0.89 [0.46–1.74]**
**Literacy**									
Cannot read			**1**	**1**		**1**	**1**		**1**
Able to read part of sentence			**0.67 [0.36–1.22]**	**0.62 [0.32–1.18]**		**0.73 [0.39–1.39]**	**0.7 [0.36–1.37]**		**0.66 [0.31–1.37]**
Able to read whole sentence			**1.06 [0.72–1.57]**	**1.13 [0.71–1.81]**		**0.89 [0.59–1.36]**	**0.97 [0.59–1.60]**		**1.04 [0.601–1.77]**
**Total children ever born**			1.18 [1.09–1.26]***	1.29 [1.17–1.42]***		1.39 [1.23–1.57]***	1.51 [1.32–1.72]***		1.49 [1.30–1.69]***
**Type of delivery**									
Normal			**1**	**1**		**1**	**1**		**1**
Caesarean			**1.32 [0.76–2.32]**	**1.41 [0.80–2.50]**		**1.15 [0.61–2.18]**	**1.21 [0.63–2.31]**		**1.09 [0.51–2.32]**
**Place of delivery**									
Facility			**1**	**1**		**1**	**1**		**1**
Home			0.64 [0.45–0.91]*	0.63 [0.43–0.90]*		**0.70 [0.47–1.04]**	**0.70 [0.46–1.07]**		**0.70 [0.45–1.09]**
**Early breastfeeding**									
Within one hour			**1**	**1**		**1**	**1**		**1**
After one hour or more			**1.16 [0.87–1.53]**	**1.13 [0.84–1.53]**		**1.01 [0.74–1.38]**	**0.99 [0.71–1.39]**		**1.06 [0.74–1.5]**
**Currently using contraceptive**									
No			**1**	**1**		**1**	**1**		**1**
Yes			0.56 [0.39–0.83]**	0.55 [0.38–0.82]**		0.54 [0.35–0.84]**	0.54 [0.34–0.85]**		0.56 [0.35–0.90]*
**Ever terminated pregnancy**									
Never			**1**	**1**		**1**	**1**		**1**
Yes			**1.18 [0.82–1.70]**	**1.13 [0.79–1.62]**		**1.32 [0.90–1.93]**	**1.27 [0.87–1.86]**		**1.24 [0.81–1.90]**
**GEOSPATIAL FACTORS**									
The length of the growing season									
< =8 months								**1**	**1**
9-10 months								**1.08 [0.64–1.8]**	**0.91 [0.49–1.72]**
11–12 months								**1.78 [0.85–3.71]**	**1.38 [0.46–4.17]**
Environmental temperature via restricted cubic spline									
**Livestock_goats**								**1.00 [1.00–1.01]**	**1.00 [1.00–1.01]**
Malaria prevalence								**0.58 [0.1–3.25]**	**0.25 [0.03–2.17]**
**Log of the population density**								**0.92 [0.79–1.09]**	0.76 [0.60–0.96]*
**Rainfall via** restricted cubic splines								**1.00 [1.00–1.01]**	**1.00 [0.99–1.00]**
**Vegetation index**									
Less than 25th percentile								**1**	**1**
25th–50th percentile								**1.61 [0.97–2.65]**	**1.19 [0.67–2.12]**
50th–75th percentile								**1.20 [0.61–2.38]**	**0.68 [0.28–1.63]**
> 75th percentile								**1.19 [0.53–2.64]**	**0.52 [0.19–1.44]**
**Proximity to a protected area**									
Less than 25th percentile								**1**	**1**
25th–50th percentile								**1.37 [0.95–1.97]**	1.83 [1.15–2.92]*
50th–75th percentile								**0.89 [0.62–1.27]**	**1.24 [0.76–2.02]**
> 75th percentile								**0.89 [0.59–1.36]**	**1.08 [0.59–1.97]**
**Survey year**									
2003	1	1	**1**	**1**	**1**	**1**	**1**	**1**	**1**
2008	0.76 [0.55–1.06]	0.73 [0.53–1.01]	**0.84 [0.6–1.18]**	**0.9 [0.61–1.34]**	**0.75 [0.52–1.07]**	**0.83 [0.58–1.2]**	**0.92 [0.59–1.42]**	0.66 [0.49–0.9]**	**0.87 [0.55–1.38]**
2014	0.71 [0.53–0.96]*	0.65 [0.48–0.86]**	0.70 [0.5–0.97]*	**0.76 [0.52–1.12]**	0.68 [0.49–0.94]*	**0.71 [0.5–1]**	**0.80 [0.53–1.22]**	0.64 [0.49–0.84]**	**0.81 [0.54–1.23]**

*Abbreviations*: *aRR* Adjusted Relative Risk. *P*-value notation: ***p < 0.001, **p < 0.01, *p < 0.05

### Risk factors of neonatal deaths: Most recent evidence from the 2017 Ghana maternal health survey

This study utilized data from 15,348 children born in the five years preceding the Maternal Health Survey conducted in 2017.

### Risk factors of neonatal mortality in 2017

A unit increase in the number of children born to a woman is associated with an approximately 26% increase in neonatal deaths [aRR = 1.26, 95% CI: 1.03–1.54, *p* < 0.05, Table 4]. However, larger household size was associated with a reduced risk of neonatal deaths by 12% [aRR = 0.88, 95% CI: 0.80–0.89, p < 0.05, Table 4]. The mothers who gave birth to two or more babies had approximately four times the risk of experiencing neonatal deaths compared to mothers who gave birth to single babies [aRR = 4.1, 95% CI: 1.9–8.9, *p* < 0.05, Table 4]. The risk of neonatal deaths among children born in rural areas is approximately two times the risk of neonatal deaths among children born in urban areas [aRR = 1.8, 95% CI: 1.1–2.9, *p* < 0.05, Table 4]. The following variables (ANC attendance, Tetanus injection) were also associated with neonatal deaths. The results must be interpreted with caution because the data originate from the most recent birth during the survey. That is, we did not adjust for other confounding factors due to the sample size. Among mothers who did not attend ANC, the risk of neonatal death was approximately five times as high as the risk of neonatal deaths for mothers who attended ANC [aRR = 4.7, 95% CI: 1.8–12.1, *p* < 0.05, Table 4]. The mothers who did not receive any tetanus injection during pregnancy were also at high risk (approximately two times) of having neonatal deaths [aRR = 2.1, 95% CI: 1.1–4.0, *p* < 0.05, Table 4]. It is important to emphasize National Health Insurance (NHI) did not provide protection against neonatal death, as shown in 2017 GMHS data. The results showed that mothers who had no valid NHI card were at a lower risk of neonatal deaths compared with mothers with valid NHI card [aRR = 0.6, 95% CI: 0.4–0.9, *p* < 0.05, Table 4]. This finding is quite surprising, but what could possibly explain the observed phenomenon could be that women who are fully aware of their high-risk status before delivery are more likely to secure a valid NHI card during the antenatal period to mitigate the cost of seeking healthcare in case of emergency compared to women who are perceived to be in good health. The results should be interpreted with caution since we did not adjust for confounding factors because the data were only available for the most recent birth reducing the sample size drastically.

**Table 4:** Risk factors of neonatal mortality in Ghana: Evidence from the 2017 Ghana maternal health survey

Variables	Neonatal mortality	
	Poisson	Negative Binomial
	aRR[95% CI]	aRR[95% CI]
Sex of the child		
*Male*	Ref	
*Female*	0.84[0.54–1.33]	0.60[0.34–1.05]
Mothers education		
*None*	Ref	
*Primary/middle*	1.03[0.53–2.02]	1.21 [0.51–2.85]
*JHS/JSS*	0.97[0.53–1.77]	1.74 [0.74–4.11]
*SHS+*	2.43[1.09–5.40] *	7.25 [2.36–22.23]**
Current age	0.95[0.89–1.01]	1.01 [0.94–1.08]
Marital status		
*Yes, currently married*	Ref	
*Yes, living with a man*	0.88[0.49–1.60]	1.00 [0.52–1.95]
*No, not in union*	1.06[0.48–2.32]	1.19 [0.45–3.09]
Parity	1.26[1.03–1.54] *	1.27 [1.01–1.59]*
Multiple birth		
*single*	Ref	
*Multiple*	4.12 [1.91–8.85] ***	7.38 [3.15–17.3]***
*Age at first sex*	0.99[0.90–1.08]	0.98 [0.88–1.09]
Place of delivery		
Hospital	Ref	
Home	1.24[0.67–2.29]	1.17 [0.51–2.67]
Household size	0.88[0.76–1.03]	0.88 [0.80–0.98]*
**Water**		
*Improved*	Ref	
*Not improved*	0.97[0.54–1.73]	1.44 [0.63–3.29]
**Sanitation**		
*Improved*	Ref	
*Not improved*	0.97[0.54–1.73]	0.51 [0.24–1.10]
**Owns agriculture land**		
*Yes*	Ref	
*No*	0.83[0.49–1.41]	0.7 [0.35–1.38]
**Owns Livestock**		
*Yes*	Ref	
*No*	1.20[0.69–2.06]	1.53 [0.78–3.00]
**Ever had an abortion**		
*Yes*	Ref	
***No***	0.77[0.44–1.34]	1.14 [0.55–2.36]
**Ever had miscarriage**		
*Yes*	Ref	
*No*	1.35[0.75–2.45]	2.16 [0.98–4.76]
**Ever had stillbirth**		
*Yes*	Ref	
*No*	0.79[0.31–2.02]	0.58 [0.17–1.95]
**Zone**		
*Coastal*	Ref	
*Middle*	1.08[0.68–1.72]	1.06 [0.55–2.06]
*Northern*	0.81[0.45–1.45]	1.58 [0.67–3.74]
Place of residence		
*Urban*	Ref	
*Rural*	1.77 [1.07–2.90]*	3.77 [1.83–7.76]***
Antenatal care^¥^		
*Yes*	Ref	
*No*	4.65[1.80–12.06] **	5.16 [2.06–12.91]***
Size at birth^¥^		
*Very large*	Ref	
*Large*	0.61[0.28–1.36]	0.57 [0.24–1.36]
*Average*	0.86[0.41–1.83]	0.77 [0.34–1.73]
*Small*	0.67[0.25–1.81]	0.59 [0.20–1.75]
*Very small*	1.23[0.46–3.26]	1.15 [0.41–3.24]
Tetanus during pregnancy^¥^		
*Yes*	Ref	
*No*	2.10[1.11–3.97] *	2.06 [1.07–3.99]*
*Iron*		
*Yes*	Ref	
*No*	2.02[0.89–4.62]	2.10 [0.88–5.01]
Fansidar (sulfadoxine and pyrimethamine) during pregnancy^¥^		
*Yes*	ref	
*No*	2.10[0.95–4.64]	2.22 [0.98–5.06]
Valid NHI card^¥^		
*Yes*	Ref	
*No*	0.58[0.37–0.91] *	0.64 [0.39–1.05]

*P*-value notation: ***p < 0.001, **p < 0.01, *p < 0.05, ^¥^ Unadjusted estimates from Poisson and Negative Binomial models since the variable was only measured for most recent birth. *Abbreviation*: *NHI* National Health Insurance

### Synthesizing all the available evidence on the factors influencing neonatal mortality in Ghana-1998-2017

This section combines all the available evidence from 1998 to 2017 to reclassify neonatal mortality predictors into major contributing factors, moderate contributing factors, and minor contributing factors. Major contributing factors are factors that are biologically plausible and have been consistent over the years in increasing mortality among neonates using the findings from the multivariable regression models. The moderate contributing factors are those that are biologically plausible, but for lack of data over the period, they were not included in the multivariable regression models to quantify the effect size. These factors are relevant, but the power of the study will be affected if included in the multivariable model because of the sample size. For instance, antenatal and postnatal care attendance are good indicators of neonatal deaths, but they were only measured for the most recent birth reducing the effective sample size drastically. The minor contributing factors are difficult to explain why and how they contribute to neonatal deaths, and further studies are needed to understand the biological underpinnings. Table 5 summarizes these contributing factors.

**Table 5:** Synthesizing all the available evidence on the factors influencing neonatal mortality in Ghana-1998-2017

Contributing factors	The extent of the contributing factors	High neonatal mortality
Multiple births (twin, triplet, etc.)	Major	Yes
Smaller household size	Major	Yes
Perceived smaller birth weight	Major	Yes
Women who are not using contraceptive	Major	Yes
A higher number of biological children (high parity)	Major	Yes
Children born in the northern sector	Major†	Yes†
Population density	Major	Yes
Birth spacing less than 24 month	Moderate	Yes
Mothers age at birth (older mothers 30+)	Moderate	Yes
Mothers who had no tetanus injection during pregnancy	Moderate	Yes
Mothers who delivered via cesarean section	Moderate	Yes
Children who did not receive vitamin A two months after delivery	Moderate	No
Mothers who did not attend antenatal care	Moderate	Yes
No access to an improved water source	Moderate	No
Children who live in rural areas	Moderate	No
Not receiving Fansidar (sulfadoxine and pyrimethamine) against malaria	Moderate	No
Household ownership of bednet	Moderate	No
Children who are Muslims were at a higher risk of death between 2003 and 2014	Minor	No
Violence against women	Minor	No
Mothers whose husband or partners have two or more wives	Minor	Yes
Shorter mothers	Minor	Yes
Mothers with SHS or higher education	Minor	Yes

^†^: Contributing factor before 2017 but the impact disappeared in the 2017 Ghana Maternal Health Survey Data Analysis

## Discussion

This study investigated the impact of several risk factors of neonatal mortality in Ghana between 1998 and 2017. It was observed that maternal and child characteristics explain a larger proportion of neonatal deaths variations compared to household and selected environmental (geospatial) factors. The following were identified to be major contributing factors to neonatal mortality: multiple births, birth spacing, birth weight perceived to be small, smaller household size, an increasing number of children born to a woman of reproductive age, not using a contraceptive, living in the northern part of the country, mothers age at childbirth, mothers who delivered via cesarean section, mothers who gave birth at the age of 30 years or more, a household with no bed net, mothers with short stature, mothers who did not receive Fansidar (sulfadoxine and pyrimethamine) during antenatal, and place of residence. The next section discusses factors that were considered major determinants of neonatal deaths in Ghana. Specifically, the discussion focused on multiple births, household size, parity, contraceptive use, birth weight, and population density.

### Multiple births

Multiple births are generally classified as high-risk birth in the medical literature because they are associated with fetal and neonatal complications that require special and expensive medical care [18]. The biological complications associated with multiple births usually lead to a greater risk of congenital disabilities and accounted for a larger percentage of prenatal deaths [19]. Besides, twins, triplets, etc., suffer lower birth weight, have intrauterine growth restriction and congenital abnormalities, and preterm delivery, which are key determinants of neonatal deaths in the early years of life. This could be explained by the fact that there could be some underlying biological complications that extend beyond the neonatal and infant years, but this has not been studied extensively in the literature. Our result showed that the risk of neonatal deaths was higher among multiple births compared to singletons, which is consistent with a previous study in sub-Saharan Africa that indicated that one-fifth of twins in the region die before age five years, three times the mortality rate among singletons [20]. There should be a significant improvement in health-service delivery during pregnancy, at delivery, and postpartum [21, 22]. It was evident that the impact of multiple births has not reduced over the period which is indicative of the fact that not much has been done about the phenomenon from the perspective of key stakeholders (mothers, Ghana Health Service, Ministry of Health, Midwives, Nurses, Doctors, and all other health practitioners). This study could not determine the proportion of multiple births attributable to assisted reproductive technology and whether the increase in the number of multiple births resulted from multiple pregnancies caused by the practice of transferring more than one embryo into the uterus during Vitro fertilization (IVF). Looking at the trend of the effect of multiple births, this study recommends routine training of midwives, doctors, and nurses in general on how to manage multiple births during antenatal, delivery, and postnatal.

### Household size

This study found that larger household size is associated with a reduced risk of child mortality. Admittedly larger household size could be associated with people living below the poverty line and competing for the same socioeconomic and health resources, increasing child mortality risk. However, the observed protective effect of larger household size could be explained by the Ghanaian culture of providing food security, financial and medical support to the needy in larger households. More research needs to be done to better understand household size dynamics and its relative effect on neonatal mortality in Ghana.

### Parity

This study found that high parity births are associated with a higher neonatal mortality than low parity births, consistent with the findings from the literature [23, 24]. High parity correlates with lower coverage of maternal and child health services such as ANC and PNC attendance and skilled birth delivery, vitamin A supplementation, vaccination and immunization, and iron supplementation that might increase mortality [25]. There is a positive correlation between birth spacing and high parity births. There should be intensive education on birth spacing and the risk associated with high parity births. Women should be encouraged to use contraceptive methods to increase birth spacing and control the number of children born to women of reproductive age.

### Contraceptive use

The use of contraceptives increases birth spacing and unwanted pregnancies reducing complications associated with shorter birth intervals. Women who used contraceptives were likely to reduce the number of births to three or fewer children alleviating the negative impact of high parity-associated with child mortality. To design and implement effective interventions aim at improving the prevalence of contraceptive use, we need to understand the drivers of low contraceptive use. There should be religious and culturally sensitive intensive education on contraceptive use among women, and these contraceptives should be readily available and affordable to the general population.

### Birth weight

Children that were perceived to be small were at a greater risk of child mortality, and the finding is consistent with what was reported previously [26, 27]. Low birth weight is associated with high preterm birth and congenital abnormalities leading to high neonatal and infant deaths. The consequences of low birth weight do not exclude long term effects as Watkins et al, 2018 [28] showed that low birth weight is associated with increased death rates not only in infancy but also through to adolescence. Improving the mother’s nutritional status and avoiding high-risk behavior such as smoking and alcohol intake during pregnancy is critical to improving birth weight.

### Cesarean section

The study showed that increased cesarean section increases the risk of neonatal deaths in Ghana, which is consistent with the studies mentioned above. This could be possible because obstetricians are more likely to advise women with birth complications to opt for a cesarean section, but on the contrary, the majority of early-term elective cesarean sections can be postponed [29]. Elective cesarean deliveries are on the rise and become more popular among expectant mothers and health professionals because cesarean delivery globally represents a potentially life-saving procedure for both mother and neonate in labor complications and health conditions of expectant mothers that require early or immediate delivery. However, in the absence of serious medical complications and the highly negligible likelihood of having stillbirth, neonatal and maternal deaths, cesarean delivery can pose avoidable short-term and near-future risks to both the mother and newborn, including birth injury, seizures, jaundice and hypoglycemia, sepsis, longer maternal recovery, neonatal respiratory problems, and potentially severe complications in subsequent pregnancies [30–32]. Studies have consistently shown an increased risk in prenatal deaths and infant mortality, though the causal pathway is not always clear [33–36]. The increasing number of unwarranted cesarean sections globally is a major concern to both healthcare providers and policymakers as stipulated in agenda “Healthy People 2020” with the goal of reducing the cesarean delivery rate by 10 % among low-risk women giving birth for the first time and among low-risk women with a prior cesarean section [37]. There should be a conscious effort to reduce the cesarean section rate among mothers who are at low risk of birth complications.

### Impact of environmental factors on neonatal mortality

The population density was the only geospatial variable associated with neonatal mortality. Higher population density is usually associated with low health service utilization and quality healthcare. However, this study found a protective effect of population density on neonatal deaths. This could be explained by the fact that higher population density in Ghana may be associated with higher maternal health coverage [38], which could reduce the incidence of neonatal deaths. Mothers who live in regions and areas with highly dispersed populations and low socioeconomic status could face a higher burden of assessing quality health healthcare.

### Strength and limitations

The co-integration of individual-level data and geospatial covariates in cross-sectional ecological studies can help researchers and policymakers understand the causal pathway of the risk of neonatal deaths in Ghana [39]. The study’s power to detect significant effect of covariates improved by using four GDHS data and one GMHS data that were collected within the MDG and post MDG period. This study applied more rigorous geospatial and non-spatial statistical models to nationally representative survey data to determine the impact of a wide-ranging set of possible correlates of neonatal deaths.

Nonetheless, there are several limitations to the study that are worth noting. First, the DHS data used for the analysis is a cross-sectional survey, and we cannot infer that the identified risk factors caused the death of the children. The study only showed that these risk factors are just associated with neonatal mortality. The true cause of death could only be determined by conducting an extensive death autopsy for all children under-five. However, the survey data used do not contain these children’s autopsy report, making it difficult to assess the cause, mode, and manner of death.

Some important predictors of neonatal deaths, such as vaccination, fever, diarrhea, nutritional and respiratory infections, were only collected for surviving children and were therefore excluded from this study.

Third, there is the possibility of recall bias as the DHS collects information from respondents about past events, behaviors, and health outcomes. For instance, information concerning women’s receipt of maternal care services such as ANC, PNC, tetanus injection, mode of delivery, etc., is all subject to recall bias. Several key indicators, such as PNC attendance, could not be studied because they were only available for the women’s most recent birth. We omitted the 1998 GDHS data set from the final model because most of the interest indicators were not measured during the survey. For instance, no variable measured household wealth in the data set, and therefore, we couldn’t have assessed the impact of socioeconomic status on neonatal deaths if we had included the 1998 GDHS in the multiple variable analysis.

### Policy implications

Through the Ministry of Health and the Ghana Health Service, the government of Ghana should organize routine practical simulation training for obstetricians, general medical practitioners, nurses, and midwives on how to handle complications associated with multiple births. There should be a clearly defined direct policy on multiple births that incorporates routine training of health professionals. There should be a policy for all health facilities to design a database that captures all multiple births in the health facility or the community, including multiple births from In vitro fertilization (IVF) intervention. This will facilitate the process of tracking these births during antenatal, delivery, postnatal through to age 5. This activity could be spearheaded by the community health nurses and monitored by a specialist obstetrician. Efforts should be made to monitor both twins, triplet etc. during labor. There should be early pregnancy ultrasound scans, and monochorionic twins should be referred for specialist obstetrician care. Except for a few private health facilities, most pregnancies, including multiple births, are generally handled by midwives at the health facility, but we recommend, in addition to the midwives, there should be specialist obstetrician care for all multiple births during antenatal, delivery, and postnatal care. The Ministry of Health, Ghana Health Service, Ghana Medical Association, and all fertility centers across the country may consider a policy of elective single embryo transfer (eSET) to reduce the chances of a woman having multiple births and to reduce to the barest minimum the complications associated with multiple births among women and their respective children who are delivered via Assisted Reproductive Technology (ART). To encourage eSET, blastocyst culture, preimplantation genetic screening, and time-lapse imaging could help identify the embryo with the greatest implantation potential. The MOH and the GHS should use the pregnancy school and the media (radio, television, and social media), together with community health nurses and other healthcare professionals, to provide intensive education about the risk of multiple births, high parity, birth spacing, antenatal care attendance, facility delivery, postnatal care, immunization, tetanus injection, contraceptive use by mothers, efficient ways to improve the weight of the unborn baby and nutritional requirements during pregnancy.

## Conclusion

This study identified several factors associated with neonatal deaths in Ghana. Some of the factors have been consistent over time, whiles the impact of other factors fluctuates and diminishes between survey years. The environmental factors did not have a significant impact on child mortality. The household, maternal and child-related factors such as household size, parity, multiple births, birth weight, place of residence, antenatal care attendance, access to improved water source among rural residence, immunization coverage, and tetanus injection were among the few factors that affected neonatal deaths in Ghana. This study recommends an integrated intervention that simultaneously addressed these challenges.

## Supplementary Information

**Additional file 1.**

## Data Availability

The data used in the study are freely available upon request from the DHS website (https://dhsprogram.com/).

## Abbreviations

AIC: Akaike Information Criterion
ANC: Antenatal Care
aRR: Adjusted Relative Risk
ART: Assisted Reproductive Technology
BIC: Bayesian Information Criterion
CS: Cesarean Section
cvAUROC: Cross-validated Area Under the Receiver Operating Characteristics Curve
EAs: Enumeration Areas
ECD: Elective Cesarean Delivery
eSET: Elective Single Embryo Transfer
EVI: Enhanced Vegetation Index
GDHS: Ghana Demographic and Health Surveys
GHS: Ghana Health Service
GIS: Geographic Information System
GMHS: Ghana Maternal Health Survey
IPTp: Intermittent Preventive Treatment
ITN: Insecticide Treated Net
MDG: Millennium Development Goals
MOH: Ministry of Health
NHI: National Health Insurance
PNC: Postnatal Care
SDG: Sustainable Development Goals
VIF: Variance Inflation Factor
WHO: World Health Organization
