# Supplementary material for: Geospatial analysis of determinants of neonatal mortality in Ghana
Source: BMC Public Health. 2021 Mar 12;21:492. doi: 10.1186/s12889-021-10473-w (PMC7953754; doi:10.1186/s12889-021-10473-w)
Supplement: Supplementary file 1 — Additional file 1. [file 12889_2021_10473_MOESM1_ESM.docx]

**Pathway to neonatal survival in Ghana: where should the focus be? Differential impact of environmental, household, child and maternal characteristics using geospatial and non-spatial modeling approach**

#### Appendix A1: The effect of household factors on neonatal deaths in Ghana: Poisson regression model adjusting for the year of survey fixed effect, sampling weight, clustering and stratification arising from the complex survey design-1998-2014.

| **Variables** | **The risk of neonatal deaths** |
| --- | --- |
|  | **aRR [95% CI]** |
| **Age of the household head: (N=16010)** | p=0.295 |
| 16-29 | ref |
| 30-39 | 1 [0.75,1.33] |
| 40-49 | 1.28 [0.92,1.77] |
| 50+ | 1.19 [0.86,1.65] |
| **The sex of the household Head: (N=16018)** | p=0.74 |
| Male | ref |
| Female | 1.04 [0.82,1.33] |
| **Place of residence: (N=16018)** | p=0.12 |
| Urban | ref |
| Rural | 1.21 [0.95,1.53] |
| **Region: (N=16018)** | p=0.49 |
| Western | ref |
| Central | 1.33 [0.85,2.07] |
| Greater Accra | 0.79 [0.47,1.34] |
| Volta | 1.2 [0.73,1.98] |
| Eastern | 1.15 [0.74,1.8] |
| Ashanti | 1.34 [0.87,2.05] |
| Brong Ahafo | 1.24 [0.74,2.07] |
| northern | 1.08 [0.69,1.68] |
| Upper West | 0.87 [0.53,1.45] |
| Upper East | 1.11 [0.67,1.82] |
| **The household has access to electricity: (N=16010)** | p=0.593 |
| No | ref |
| Yes | 1.06 [0.85,1.32] |
| **The main floor material: (N=16012)** | p=0.364 |
| Cement | ref |
| Other | 0.9 [0.72,1.13] |
| **The main roofing material: (N=8876)** | p=0.824 |
| Asbestos | ref |
| Other | 1.23 [0.61,2.51] |
| Metal | 0.98 [0.63,1.54] |
| **The main wall material: (N=8876)** | p=0.774 |
| Cement | ref |
| Dirt | 0.94 [0.57,1.54] |
| Bamboo/stone with mud | 1.04 [0.67,1.62] |
| Cement blocks | 0.81 [0.49,1.33] |
| Others | 1.01 [0.64,1.6] |
| **Household access to an improved water source: (N=16017)** | p=0.115 |
| Improved | ref |
| Unimproved | 1.25 [0.95,1.65] |
| **Household has improved sanitation facility: (N=12617)** | p=0.229 |
| Not improved | ref |
| Improved, shared | 0.91 [0.63,1.32] |
| Improved, not shared | 0.74 [0.49,1.12] |
| **Household size: (N=16018)** | p=0.053 |
| Fewer | ref |
| 5-7 | 0.78 [0.62,1]* |
| 8+ | 0.71 [0.51,0.98]* |
| **Wealth index: (N=12720)** | p=0.538 |
| Poorest | ref |
| Poorer | 0.92 [0.64,1.31] |
| Middle | 1.21 [0.85,1.71] |
| Richer | 0.97 [0.66,1.42] |
| Richest | 1.07 [0.74,1.57] |

P-value notation: ***p<0.001, **p<0.01, *p<0.05. Abbreviations: ref: reference category

#### Appendix A1: The effect of child characteristics on neonatal deathsin Ghana: Poisson regression model adjusting for the year of survey fixed effect, sampling weight, clustering and stratification arising from the complex survey design-1998-2014.

| **Variables** | **The risk of neonatal deaths** |
| --- | --- |
|  | **aRR [95% CI]** |
| **The sex of the child: (N=16018)** | p=0.12 |
| Male | ref |
| Female | 0.85 [0.69,1.04] |
| **Mutiple birth: (N=16018)** | p<0.001*** |
| Single birth | ref |
| Multiple births | 4.59 [3.25,6.49]*** |
| **Birth order: (N=16018)** | p=0.015* |
| 1st child | ref |
| 2nd child | 0.6 [0.43,0.83]** |
| 3rd child | 0.93 [0.67,1.29] |
| 4th+ child | 0.98 [0.76,1.26] |
| **Birth spacing: (N=16018)** | p<0.001*** |
| First child | ref |
| <24 months | 1.66 [1.19,2.3]** |
| 24-35 months | 0.83 [0.61,1.12] |
| 36+ months | 0.67 [0.51,0.87]** |
| **Perceived birth weight: (N=15891)** | p<0.001*** |
| Very large | ref |
| Large | 0.84 [0.58,1.2] |
| Average | 0.88 [0.61,1.26] |
| Small | 1.71 [1.18,2.46]** |
| **Measured birth weight: (N=16018)** | p=0.867 |
| Norma | ref |
| Low birthweight | 0.95 [0.5,1.79] |
| **Antenatal care attendance: (N=12256)** | p=0.189 |
| No ANC visits | ref |
| 1-3 visits | 1.09 [0.64,1.86] |
| 4+ visits | 0.81 [0.51,1.31] |
| **Place of delivery: (N=15966)** | p=0.043* |
| Facility | ref |
| Home | 0.8 [0.64,0.99]* |
| **Currently breastfeeding: (N=16018)** | p<0.001*** |
| Not breastfeeding | ref |
| Breastfeeding | 0.66 [0.53,0.83]*** |
| **Tetanus injection: (N=12349)** | p=0.086 |
| No tetanus injection | ref |
| One injection | 0.86 [0.58,1.26] |
| 2+ injection | 0.68 [0.48,0.98]* |
| **Type of delivery: (N=15962)** | p=0.003** |
| Normal | ref |
| cesarean | 1.74 [1.21,2.5]** |
| **Early breastfeeding: (N=12485)** | p=0.165 |
| Within one hour | ref |
| After one hour or more | 1.22 [0.92,1.62] |

P-value notation: ***p<0.001, **p<0.01, *p<0.05. Abbreviations: ref: reference category

#### Appendix A1: The effect of maternal factors on neonatal deaths in Ghana: Poisson regression model adjusting for the year of survey fixed effect, sampling weight, clustering and stratification arising from the complex survey design-1998-2014.

| **Variables** | **The risk of neonatal deaths** |
| --- | --- |
|  | **aRR [95% CI]** |
| **Mothers age at first-child: (N=16018)** | p=0.044* |
| <18 | ref |
| 18-29 | 0.95 [0.75,1.21] |
| 30+ | 1.85 [1.08,3.19]* |
| **Religion: (N=16011)** | p=0.968 |
| christian | ref |
| Islam | 0.96 [0.7,1.32] |
| Others | 0.96 [0.6,1.55] |
| **Ethnicity: (N=16017)** | p=0.354 |
| Akan | ref |
| Mole-dagbani | 0.82 [0.6,1.11] |
| Others | 1.02 [0.8,1.31] |
| **The current marital status of the mother: (N=16018)** | p=0.808 |
| Never married | ref |
| Married | 0.9 [0.65,1.25] |
| Not married but living together | 0.89 [0.6,1.33] |
| **Total children ever born: (N=16018)** | p=0.003** |
| 1 | ref |
| 2 | 0.94 [0.66,1.35] |
| 3 | 1.02 [0.7,1.49] |
| 4 | 0.98 [0.62,1.53] |
| 5 | 1.55 [1.11,2.15]** |
| **Number in co-wives: (N=15316)** | p=0.009** |
| One | ref |
| More than one | 1.38 [1.08,1.77]** |
| **Age at first sex: (N=15475)** | p=0.819 |
| 8-17 | ref |
| 18-24 | 0.98 [0.79,1.21] |
| **Mothers education level: (N=16018)** | p=0.328 |
| No education | ref |
| Primary | 1.12 [0.9,1.4] |
| Higher | 0.73 [0.36,1.5] |
| **Literacy: (N=12679)** | p=0.507 |
| cannot read | ref |
| Able to read part of sentence | 0.77 [0.5,1.2] |
| Able to read whole sentence | 0.95 [0.69,1.3] |
| **Wanted pregnancy when became pregnant: (N=15989)** | p=0.425 |
| Then | ref |
| Later | 0.84 [0.64,1.1] |
| No more | 0.91 [0.63,1.3] |
| **Ever terminated pregnancy: (N=16012)** | p=0.063 |
| Never | ref |
| Yes | 1.27 [0.99,1.64] |
| **Currently using contraceptive: (N=16018)** | p=0.055 |
| No | ref |
| Yes | 0.75 [0.55,1.01]** |
| **Obesity among mothers: (N=12881)** | p=0.975 |
| Underweight | ref |
| Normal | 1.02 [0.64,1.61] |
| Overweight | 0.95 [0.58,1.55] |
| Obese | 1.05 [0.58,1.88] |
| **Stature of the woman: (N=12940)** | p=0.003** |
| Short | ref |
| Moderate | 1.05 [0.8,1.39] |
| Tall | 0.66 [0.49,0.89]** |
| **Ideal number of children: (N=15535)** | p=0.366 |
| 0-3 | ref |
| 4-7 | 1.20 [0.91,1.59] |
| 8+ | 1.05 [0.69,1.61] |

P-value notation: ***p<0.001, **p<0.01, *p<0.05. Abbreviations: ref: reference category.

#### Appendix A1: The effect of maternal factors on neonatal deaths in Ghana: Poisson regression model adjusting for the year of survey fixed effect, sampling weight, clustering and stratification arising from the complex survey design-1998-2014.

| **Heard about family planning method: (N=16018)** | p=0.444 |
| --- | --- |
| not heard | ref |
| heard | 1.19 [0.76,1.87] |
| **Hypertensive: (N=5874)** | p=0.354 |
| No | ref |
| Yes | 1.39 [0.69,2.77] |
| **Own a house alone or jointly: (N=5883)** | p=0.663 |
| Does not own house | ref |
| Alone or jointly | 0.89 [0.52,1.52] |
| **Own a land alone or jointly: (N=5882)** | p=0.054 |
| Does not own a land | ref |
| Alone or jointly | 1.6 [0.99,2.58] |
| **Level of autonomy: (N=8876)** | p=0.695 |
| No autonomy | ref |
| Low autonomy | 0.89 [0.58,1.37] |
| Moderate autonomy | 1.13 [0.72,1.78] |
| High autonomy | 1.06 [0.66,1.69] |
| **Support any form violence against women (N=12716)** | p=0.174 |
| No | ref |
| Yes | 1.17 [0.93,1.46] |
| **Antenatal care message (N=5883)** | p=0.118 |
| 1. no | ref |
| 2. yes | 0.67 [0.4,1.11] |
| **Number of times-vegetables were eaten in the past 7 days (N=5876)** | p=0.843 |
| Never | ref |
| 1-3 times | 1.02 [0.47,2.21] |
| 4-6 times | 0.88 [0.39,1.97] |
| 7+ times | 0.82 [0.38,1.79] |
| **Number of times fruits were eaten in the past 7 days (N=5876)** | p=0.18 |
| Never | ref |
| 1-3 times | 0.95 [0.51,1.75] |
| 4-6 times | 1.66 [0.8,3.45] |
| 7+ times | 0.89 [0.47,1.69] |
| **Husband/partner’s occupation: (N=15186)** | p=0.547 |
| Professional | ref |
| Other | 0.73 [0.47,1.13] |
| Agriculture | 0.80 [0.55,1.16] |
| Skilled manual | 0.70 [0.46,1.09] |
| Unskilled manual | 0.69 [0.33,1.47] |
| **Husband age in years: (N=14186)** | p=0.402 |
| 17-29 | ref |
| 30-39 | 0.82 [0.61,1.11] |
| 40-49 | 0.97 [0.68,1.37] |
| 50+years | 0.78 [0.52,1.19] |
| **Husband education level: (N=14757)** | p=0.312 |
| No education | ref |
| Primary | 1.31 [0.91,1.91] |
| Secondary | 0.99 [0.76,1.29] |
| Higher | 1.31 [0.82,2.09] |

P-value notation: ***p<0.001, **p<0.01, *p<0.05. Abbreviations: ref: reference category

#### Appendix A1: The effect of coverage of health intervention on neonatal deaths in Ghana: Poisson regression model adjusting for the year of survey fixed effect, sampling weight, clustering, and stratification arising from the complex survey design-1998-2014.

| **Variables** | **The risk of neonatal deaths** |
| --- | --- |
|  | **uRR [95% CI]** |
| **NHI: (N=8870)** | p=0.059 |
| Not insured | ref |
| Insured | 1.36 [0.99,1.87] |
| **Household sprayed: (N=5844)** | p=0.639 |
| No | ref |
| Yes | 1.13 [0.69,1.85] |
| **Number of net: (N=12720)** | p=0.741 |
| No net | ref |
| One | 0.96 [0.7,1.33] |
| Two | 0.83 [0.55,1.24] |
| 3+ | 1.05 [0.69,1.58] |
| **Sleep under bednet: (N=12720)** | p=0.228 |
| No | ref |
| Yes | 0.85 [0.65,1.11] |
| **Heard any malaria message: (N=16018)** | p=0.591 |
| Less than 7 times | ref |
| Seven or more times | 0.9 [0.61,1.33] |
| **Over-crowding (N=12174)** | p=0.878 |
| >2 persons per room | ref |
| <=2 persons per room | 1.02 [0.78,1.33] |
| **Vitamin A: (N=9138)** | p<0.001*** |
| No | ref |
| Yes | 0.11 [0.07,0.19]*** |
| **Pregnant women program: (N=5883)** | p=0.116 |
| 0. No | ref |
| 1. Yes | 1.46 [0.91,2.33] |
| **Programs helping children under 18 accessing health service (N=5883)** | p=0.113 |
| No | ref |
| Yes | 1.41 [0.92,2.16] |
| **Health facility visit in past 6 months: (N=5884)** | p=0.349 |
| No | ref |
| Yes | 1.23 [0.8,1.88] |
| **Have bednet: (N=12720)** | p=0.669 |
| No net | ref |
| Have net | 0.94 [0.7,1.26] |
| **Survey year: (N=16018)** | p=0.005** |
| 1998 | ref |
| 2003 | 1.48 [1.09,2.02]* |
| 2008 | 1.04 [0.74,1.46] |
| 2014 | 0.95 [0.69,1.3] |

P-value notation: ***p<0.001, **p<0.01, *p<0.05. Abbreviations: ref: reference category, NHI: National Health Insurance.

## Testing of spatial autocorrelation among the geospatial covariates: Moran I index

The Table in Appendix B1 below shows the test to determine whether there is spatial autocorrelation among the variables. We tested the extent to which regions in Ghana are interdependent (global spatial autocorrelation) and further tested the extent to which certain clusters (hotspots) showed a high spatial autocorrelation (local spatial autocorrelation). Since the focus was on global autocorrelation, we present the results on global autocorrelation in appendix B1. The Table in Appendix B1 shows the Moran I of the null hypothesis of no global or local spatial autocorrelation for each of the geospatial covariate that was studied. The results showed that there was no global spatial autocorrelation among the covariates as none of the p-values was found to be statistically significant (Appendix B1). This indicates that there was no need to use specific geospatial models that account for spatial autocorrelation. We, therefore, relied on the traditional regression models to estimate the parameter of interest in all the 9 regression but the complex survey design features (clustering, weighting, and stratification) were adjusted for in all our regression models.

#### Appendix B1: The Moran’s I test for spatial autocorrelation

| Spatial covariates | **Neonatal** |
| --- | --- |
|  | **Chi, P-value** |
| Global Human Footprint | 0.84,0.3606 |
| Gross Cell Production | 0.20,0.6553 |
| Growing Season Length | 0.30,0.5825 |
| Livestock Cattle | 0.34,0.5613 |
| Livestock Chickens | 0.26,0.6076 |
| Livestock Goats | 0.12,0.7324 |
| Livestock Pigs | 0.31,0.5778 |
| Livestock Sheep | 0.33,0.5684 |
| Slope | 0.21,0.6459 |
| Irrigation | 0.35,0.5536 |
| Population Count 2005 (Number of People) | 0.31,0.5751 |
| Population Count 2010 (Number of People) | 0.31,0.5750 |
| Population Count 2015 (Number of People) | 0.31,0.5749 |
| All Population Density (Number of People Per Square Kilometer) | 0.31,0.5750 |
| Precipitation | 0.31,0.5747 |
| Aridity | 0.32,0.5700 |
| Built Population | 0.51,0.4762 |
| Surface Temperature | 0.36,0.5476 |
| Diurnal Temperature | 0.15,0.6948 |
| Enhanced Vegetation Index | 0.33,0.5657 |
| ITN Coverage (Number of People) | 0.60,0.4400 |
| Land Surface Temperature | 0.34,0.5588 |
| Malaria Incidence (Number of People Per Year) | 0.41,0.5234 |
| Mala Prevalence (Number of People Per Year) | 0.39,0.5323 |
| Maximum Temperature | 0.31,0.5790 |
| Mean Temperature | 0.28,0.5969 |
| Minimum Temperature | 0.01,0.9259 |
| All Nightland Temperature | 0.29,0.5876 |
| Potential Evapotranspiration (Millimeters Per Year) | 0.33,0.5637 |
| Proximity to National Borders (Meters) | 0.34,0.5616 |
| Proximity to Protected Areas | 0.01,0.9170 |
| Proximity to Water | 0.36,0.5495 |
| Rainfall (Millimeters Per Year) | 0.25,0.6151 |
| Environmental Temperature | 0.33,0.5666 |
| Travel Time (Hours) | 0.53,0.4652 |
| All U5 Pop | 0.16,0.6899 |
| UN Population Count (Number Of People) | 0.32,0.5698 |
| UN Population Density | 0.17,0.6822 |
| Wet Days | 0.32,0.5690 |

Abbreviations: UN-United Nations

## Testing for multicollinearity of the Geospatial covariates

Appendix B2 shows the multicollinearity estimates for all the geospatial covariates. We dropped all variables with a multicollinearity index of 10 or more and also dropped variables that were already captured as part of the household characteristics. For instance, ITN use was dropped from the multivariable analysis because a question was asked about household ownership of bed-net in the household data. Thus our geospatial model and all other subsequent models that involve geospatial covariates were based on the following geospatial variables: Length of the growing season in month (categorized as less or equal to 8 months, 9-10 months, 11 or months), environmental temperature (modeled as a four-knot restricted cubic splines), rainfall (modeled as four not restricted cubic splines), population density (log-transformed), vegetation index areas (modeled using the quartile distribution), proximity to protected areas (modeled using the quartile distribution), malaria prevalence, livestock (goat).

#### Appendix B2: Checking for multicollinearity of the geospatial covariates using the variance inflation factor.

| Variable | VIF | 1/VIF |
| --- | --- | --- |
| All population | 4.11 | 0.243602 |
| Precipitation | 141.24 | 0.00708 |
| Aridity | 431.91 | 0.002315 |
| Built population | 10.04 | 0.099564 |
| Surface temperature | 639.56 | 0.001564 |
| Diurnal temperature | 24332.26 | 0.000041 |
| Enhanced vegetation index | 13.88 | 0.072066 |
| ITN coverage | 5.84 | 0.17134 |
| Land surface temperature | 878.81 | 0.001138 |
| Malaria incidence | 28.09 | 0.035601 |
| Mala prevalence | 24.1 | 0.041491 |
| Maximum temperature | 129710 | 0.000008 |
| Mean temperature | 48325.06 | 0.000021 |
| Nightlights | 52.44 | 0.01907 |
| Potential Evapotranspiration (pet) | 154.46 | 0.006474 |
| Proximity to Protected Areas (meters) | 7.37 | 0.135711 |
| Proximity to National Borders | 30.83 | 0.032437 |
| Environmental Temperature | 12.68 | 0.078864 |
| Travel time | 2.31 | 0.432472 |
| Under 5 population | 2.04 | 0.491111 |
| UN Population Count | 6.62 | 0.151147 |
| Un population density | 7.37 | 0.135627 |
| Wet days | 30.1 | 0.033228 |
| Growing season length | 17.25 | 0.057966 |
| Gross Cell Production | 5.81 | 0.172007 |
| Drought episodes |  |  |
| 2 | 20.61 | 0.048525 |
| 3 | 17.49 | 0.057171 |
| 4 | 6.58 | 0.152081 |
| Mean VIF | 7318.53 |  |

Abbreviation: VIF-Variance Inflation Factor.

**The distribution of geospatial covariates**

All the covariates with substantial differences between the mean and the median were log-transformed in all subsequent analyses. For instance, the United Nations population density was heavily skewed and they were log-transformed. Appendix 3 below shows the spatial averages of the geospatial covariates that were studied.

#### Appendix 3: The distribution of the geospatial covariates

| **Variable** | **Mean (SD)** | **Minimum** | **Maximum** | **Median (iqr)** |
| --- | --- | --- | --- | --- |
| Aridity | 27(5) | 17 | 41 | 27(7.4) |
| Built Population | 0.24(0.34) | 0 | 1 | 0.038(0.4) |
| Enhanced Vegetation Index | 3317(1035) | 0 | 5023 | 3352(1870) |
| Global Human Footprint | 44(20) | 16 | 100 | 35(27) |
| ITN Coverage | 0.28(0.098) | 0.09 | 0.49 | 0.29(0.13) |
| Nightlights Composite | 18(2.2) | 0.25 | 29 | 18(2) |
| Precipitation | 92(8.7) | 78 | 126 | 92(13) |
| Proximity to National Borders | 63917(54048) | 38 | 184907 | 48935(89680) |
| Proximity to Protected Areas (meters) | 55810(33984 | 0 | 149696 | 52879(46918) |
| Proximity to water (meters) | 73001(70376) | 0 | 271913 | 44928(94940) |
| Rainfall | 1193(209) | 724 | 1771 | 1227(341) |
| Growing season length-Mean | 10(1.5) | 7 | 12 | 11(3) |
| Environmental Temperature | 27(0.92) | 23 | 29 | 27(1.3) |
| Travel Times | 83(75) | 0.86 | 499 | 71(106) |
| Slope | 0.5(0.6) | 0 | 4.2 | 0.3(0.53) |
| UN Population Density | 1353(2813) | 9.3 | 10477 | 160(402) |
| Potential Evapotranspiration (pet) | 3.5(0.41) | 3 | 4.5 | 3.3(0.48) |
| livestock cattle | 5.2(11) | 0 | 170 | 0.48(2.4) |
| livestock chickens | 298(1087) | 0 | 17227 | 68(136) |
| livestock goats | 23(111) | 0 | 3226 | 3.5(17) |
| livestock pigs | 3.8(13) | 0 | 182 | 1.1(2.8) |
| livestock sheep | 15(76) | 0 | 2569 | 5.2(16) |
| New irrigation 1 | 0.54(0.5) | 0 | 1 | 1(1) |
| New irrigation 2 | 0.46(0.5) | 0 | 1 | 0(1) |
| Wet days | 13(2.2) | 7.6 | 16 | 13(3.5) |
| Maximum temperature | 32(1.3) | 31 | 35 | 32(1.8) |
| Mean temperature | 28(0.65) | 26 | 29 | 27(0.95) |
| Minimum temperature | 23(0.82) | 21 | 25 | 23(1.1) |
| Malaria incidence | 0.4(0.12) | 0.13 | 0.6 | 0.42(0.18) |
| Malaria prevalence | 0.43(0.17) | 0.11 | 0.75 | 0.45(0.26) |
| Diurnal temperature | 9.3(1.7) | 6.8 | 12 | 9.4(2.5) |
| Surface temperature | 30(3.1) | 24 | 37 | 30(5.5) |
| Land surface temperature | 26(2) | 22 | 29 | 25(4.1) |
| Under 5_population | 198(382) | 0.057 | 2148 | 29(138) |
| All population | 61171(107769) | 49 | 1300610 | 30749(59419) |

**Appendix 4**

**Definitions of geospatial covariates**

### Aridity

The average aridity index which measures the level of humidity in the environment was obtained were obtained from the cells whose centroid falls within a radius of 10 km (for rural points) or 2 km (for urban points). Aridity was calculated by dividing the actual evapotranspiration by the potential evapotranspiration. The aridity index closer to 1.0 reflect a more humid environment. The aridity index ranges between 0.01 (Hyper Arid) and 0.99 (Humid). The data were obtained from climate data using between the periods of 1960-1990.

### Built Population

The built-up index measures the level of urbanicity associated with a particular geographic area. The study used an average built-up index of the cells whose centroid falls within a radius of 10 km (for rural points) or 2 km (for urban points). The built-up index ranges from 0.00-1.00 with figures closer to zero indicating extremely rural and 1.00 reflective of an extremely urban area: We used the index for 1990, 2000, or 2014.

### Drought Episodes

### Take it from the multivariable analysis because of a few observations

This measures the level of drought in a geographic location. The study used the average of the drought episodes indices of the cells whose centroid falls within a radius of 10 km (for rural points) or 2 km (for urban points). Individual classes between 1 (Low Drought) and 10 (High Drought). The information was obtained from the 1980-2000 precipitation data. de Sherbinin (2011) found that, among other geographic and socio-economic variables, the prevalence of drought significantly correlated with malnutrition in sub-Saharan Africa.

### Enhanced Vegetation Index

This measures the density of green leaves within a specified geographic region. The vegetation index ranges from 0-100 with zero vegetation index indicating the least vegetation and 10000 (most vegetation). The enhanced vegetation index was calculated by measuring the density of green leaves in the near-infrared and visible bands. The study used an average enhanced vegetation index of the cells whose centroid falls within a radius of 10 km (for rural points) or 2 km (for urban points). The information was obtained for 1985, 1990, 1995, 2000, 2005, 2010, or 2015. Vegetation indices such as the Enhanced Vegetation Index (EVI) are considered proxies of vector habitat; disease transmission and physical environment are strongly correlated with disease environments in determining the intensity of exposure to infectious diseases or providing favorable habitats for disease vectors (Tottrup et al. 2009). Tottrup et al. (2009) also found that districts with dense vegetation, high rainfall, and low elevation experienced the lowest reductions in child mortality. Finally, vegetation was associated with the intensity of poverty in West Africa (Sedda et al. 2015).

### Global Human Footprint

This index measures the amount of productive land appropriated on average by each person in a specific geographic location for food, water, transport, housing, waste management, and other purposes. We relied on data from 1995-2004 data. The study used the average global human footprint index of the cells whose centroid falls within a radius of 10 km (for rural points) or 2 km (for urban points). The Global human footprint index of 0 indicates an extremely rural location and 100 (extremely urban)

### ITN Coverage

The average number of people who slept under an insecticide-treated net the night before they were surveyed was used as a proxy to measure the intrinsic zeal to sleep under a bednet. The indicator was surveyed within the cells whose centroid falls within a radius of 10 km (for rural points) or 2 km (for urban points).

### Malaria

The average number of people per year who show clinical symptoms of *plasmodium falciparum* malaria within the cells whose centroid falls within a radius of 10 km (for rural points) or 2 km (for urban points). A clinical case is defined as a malaria-attributable febrile episode (body temperature in excess of 37.5 C), typically accompanied by headaches, nausea, excess sweating and/or fatigue, censored by a 30-day window (i.e., multiple bouts of symptoms

occurring within the same 30-day period are counted as a single episode). The data were available for the following years: 2000, 2005, 2010, or 2015.

### Nightlights composite

We obtained data for 2015. This measures night-time brightness which correlates with the level of human activity and socio-economic indicators. The average radiance of the cells whose centroid falls within a radius of 10 km (for rural points) or 2 km (for urban points). The nightlights intensity reflects population agglomerations and urbanicity, and can, therefore, capture urban-rural differences as well as different levels of wealth and access [1].

### Proximity to national borders

This measures how close people in a geographic area are to a national border. It is a measure of straight-line distance to the nearest international border. We obtained data for 2014.

### Proximity to protected areas (meters)

This is a straight-line distance to the nearest protected area and data were obtained for 2017.

### Proximity to water (meters)

This is a straight-line distance to the nearest major water body, and data were obtained for 2017.

### Rainfall

The average rainfall in millimeters per year of the cells whose centroid falls within a radius of 10 km (for rural points) or 2 km (for urban points). Data were obtained from 1985, 1990, 1995, 2000, 2005, 2010, 2015. Together with raised temperatures, rainfall shortage raised diarrheal prevalence in dry seasons (Bandyopadhyay, Kanji, and Wang 2012). Dos Santos and Henry (2008) also found a relationship between rainfall and neonatal deaths in Burkina Faso, especially in specific agro-climatic regions. Rainfall is associated with higher malaria burden.

### Growing season length

This measures the Length of the available growing period. It refers to the number of days within the period of temperatures above 5°C when moisture conditions are considered adequate. This was based on data collected between 1961 and 1991. Under rain-fed conditions, the beginning of the growing period is linked to the start of the rainy season. The growing period for most crops continues beyond the rainy season and, to a greater or lesser extent, crops mature on moisture stored in the soil profile. Balk et al. (2003) found that children living in areas with the shortest growing seasons, classified as arid and semiarid, had a higher risk of death. Curtis and Hossein (1998) found that aridity and length of growing season affect child malnutrition.

### Environmental temperature

The average temperature of the cells whose centroid falls within a radius of 10 km (for rural points) or 2 km (for urban points). The average temperature from the month of January to December in degrees Celsius was obtained between 1970 to 2000. Higher environmental temperature correlates with a higher diarrheal prevalence in dry seasons in sub-Saharan Africa [2].

### Travel times

The amount of time in hours it takes to reach a settlement of 50,000 or more people was obtained for the year 2000. The average travel time of the cells whose centroid falls within a radius of 10 km (for rural points) or 2 km (for urban points) was used. It is well known that populations living in remote and isolated areas are typically poorer [3] and have lower access to population centers, resources, and vaccination coverage [4]

### Slope

Topographic spatial data (elevation, slope, and roughness) are usually used as proxies for natural barriers to geographical access to city centers, markets, and health care [5], and for different exposures to infectious diseases, given the spatial variability of malaria prevalence [6]. Although [7] did not find any relationship, PJTf Webb [8], [9], found an association between topographic conditions and child stunting elevation and malnutrition.

### United Nations population density

This is the number of people per square kilometer. Data were obtained for the following years: 2000, 2005, 2010, or 2015**:** The average population density in the cells whose centroid falls within a radius of 10 km (for rural points) or 2 km (for urban points). There is a high correlation between population density (overcrowding, slum areas) and the transmission of diseases [10] and the risk of neonatal deaths increases in areas with high population density.

### Livestock density

The relationship between livestock density (ownership of livestock such as goat, sheep, cow, pig, etc) and neonatal deaths is not well understood in the medical literature and the relationship may be bidirectional. The ownership of livestock is associated with rural residence, where households depend on agriculture for part of their livelihood [11] and may contribute to their nutrition and improved health outcomes[12, 13] but a more recent study conducted in Western Kenya by Mosites, Emily, SM Thumbi, E Otiang, TF McElwain, M Njenga, PM Rabinowitz, A Rowhani-Rahbar, ML Neuhouser, S May, et al. [14] did not find statistically significant relationships between ownership and child growth.

1. Xie M, Jean N, Burke M, Lobell D, Ermon S: **Transfer learning from deep features for remote sensing and poverty mapping**. In: *Thirtieth AAAI Conference on Artificial Intelligence: 2016*; 2016.

2. Bandyopadhyay S, Kanji S, Wang LJAG: **The impact of rainfall and temperature variation on diarrheal prevalence in Sub-Saharan Africa**. 2012, **33**:63-72.

3. Tatem AJ, Gething PW, Smith DL, Hay SIJMj: **Urbanization and the global malaria recession**. 2013, **12**(1):133.

4. Metcalf CJE, Tatem A, Bjornstad ON, Lessler J, O'REILLY K, Takahashi S, Cutts F, Grenfell BTJE, Infection: **Transport networks and inequities in vaccination: remoteness shapes measles vaccine coverage and prospects for elimination across Africa**. 2015, **143**(7):1457-1466.

5. Ombok M, Adazu K, Odhiambo F, Bayoh N, Kiriinya R, Slutsker L, Hamel MJ, Williamson J, Hightower A, Laserson KFJTM *et al*: **Geospatial distribution and determinants of child mortality in rural western Kenya 2002–2005**. 2010, **15**(4):423-433.

6. Gemperli A, Vounatsou P, Kleinschmidt I, Bagayoko M, Lengeler C, Smith TJAJoE: **Spatial patterns of infant mortality in Mali: the effect of malaria endemicity**. 2004, **159**(1):64-72.

7. De Sherbinin AJP, Space, Place: **The biophysical and geographical correlates of child malnutrition in Africa**. 2011, **17**(1):27-46.

8. Webb PJTf: **Isolating Hunger: Reaching People in Need Beyond the Mainstream**. 1998.

9. Balk D, Storeygard A, Levy M, Gaskell J, Sharma M, Flor RJFP: **Child hunger in the developing world: An analysis of environmental and social correlates**. 2005, **30**(5-6):584-611.

10. Balk D, Pullum T, Storeygard A, Greenwell F, Neuman MJP, Space, Place: **A spatial analysis of childhood mortality in West Africa**. 2004, **10**(3):175-216.

11. Rogers D, Wint G, Alexander N, Pozzi F, Robinson TP: **Wealth index mapping in the Horn of Africa**. 2011.

12. Randolph TF, Schelling E, Grace D, Nicholson CF, Leroy J, Cole D, Demment M, Omore A, Zinsstag J, Ruel MJJoas: **Invited review: Role of livestock in human nutrition and health for poverty reduction in developing countries**. 2007, **85**(11):2788-2800.

13. Mosites EM, Rabinowitz PM, Thumbi SM, Montgomery JM, Palmer GH, May S, Rowhani-Rahbar A, Neuhouser ML, Walson JLJPO: **The relationship between livestock ownership and child stunting in three countries in Eastern Africa using national survey data**. 2015, **10**(9):e0136686.

14. Mosites, Emily, Thumbi SM, Otiang E, McElwain TF, Njenga M, Rabinowitz PM, Rowhani-Rahbar A, Neuhouser ML, May S *et al*: **Relations between household livestock ownership, livestock disease, and young child growth**. 2016, **146**(5):1118-1124.
